# Adaptive Immune Response to Vaccinia Virus LIVP Infection of BALB/c Mice and Protection against Lethal Reinfection with Cowpox Virus

**DOI:** 10.3390/v13081631

**Published:** 2021-08-17

**Authors:** Sergei N. Shchelkunov, Alexander A. Sergeev, Stanislav N. Yakubitskiy, Ksenia A. Titova, Stepan A. Pyankov, Irina V. Kolosova, Ekaterina V. Starostina, Mariya B. Borgoyakova, Alexey M. Zadorozhny, Denis N. Kisakov, Irina S. Shulgina, Larisa I. Karpenko

**Affiliations:** State Research Center of Virology and Biotechnology VECTOR, Rospotrebnadzor, 630559 Koltsovo, Russia; sergeev_ala@vector.nsc.ru (A.A.S.); yakubitskiy_sn@vector.nsc.ru (S.N.Y.); titova_ka@vector.nsc.ru (K.A.T.); pyankov_sa@vector.nsc.ru (S.A.P.); kolosova_iv@vector.nsc.ru (I.V.K.); starostina_ev@vector.nsc.ru (E.V.S.); borgoyakova_mb@vector.nsc.ru (M.B.B.); lexazzador@mail.ru (A.M.Z.); def_2003@mail.ru (D.N.K.); Irinash1224@icloud.com (I.S.S.); lkarpenko@ngs.ru (L.I.K.)

**Keywords:** poxviruses, vaccinia virus, vaccines, adaptive immune response, antibodies, T cells, protection

## Abstract

Mass vaccination has played a critical role in the global eradication of smallpox. Various vaccinia virus (VACV) strains, whose origin has not been clearly documented in most cases, have been used as live vaccines in different countries. These VACV strains differed in pathogenicity towards various laboratory animals and in reactogenicity exhibited upon vaccination of humans. In this work, we studied the development of humoral and cellular immune responses in BALB/c mice inoculated intranasally (i.n.) or intradermally (i.d.) with the VACV LIVP strain at a dose of 10^5^ PFU/mouse, which was used in Russia as the first generation smallpox vaccine. Active synthesis of VACV-specific IgM in the mice occurred on day 7 after inoculation, reached a maximum on day 14, and decreased by day 29. Synthesis of virus-specific IgG was detected only from day 14, and the level increased significantly by day 29 after infection of the mice. Immunization (i.n.) resulted in significantly higher production of VACV-specific antibodies compared to that upon i.d. inoculation of LIVP. There were no significant differences in the levels of the T cell response in mice after i.n. or i.d. VACV administration at any time point. The maximum level of VACV-specific T-cells was detected on day 14. By day 29 of the experiment, the level of VACV-specific T-lymphocytes in the spleen of mice significantly decreased for both immunization procedures. On day 30 after immunization with LIVP, mice were infected with the cowpox virus at a dose of 46 LD_50_. The i.n. immunized mice were resistant to this infection, while 33% of i.d. immunized mice died. Our findings indicate that the level of the humoral immune response to vaccination may play a decisive role in protection of animals from orthopoxvirus reinfection.

## 1. Introduction

By 1977, the use of vaccinia virus (VACV) as a live smallpox vaccine and strict epidemiological surveillance enabled the global eradication of such a serious human infection as smallpox. Following the certification of this historic event, the World Health Organization in 1980 strongly recommended all countries to stop smallpox vaccination [1]. This was related to the fact that mass vaccination with VACV caused severe adverse reactions in a small percentage of cases, sometimes resulting in deaths [2,3]. Over the years since then, the human population has almost lost immunity not only to smallpox but also to other zoonotic orthopoxvirus infections caused by monkeypox, cowpox, and buffalopox viruses closely related to variola (smallpox) virus. This has increased the risk of zoonotic orthopoxvirus infections to humans. Every year, more and more massive outbreaks of orthopoxvirus infections in humans have occurred in different continents [4,5,6,7]. To prevent transition of these outbreaks into widespread epidemics, methods of their immunological prophylaxis should be developed, the main of which is vaccination. Extensive use of the classic live VACV-based vaccine is currently contraindicated due to its high reactogenicity. Therefore, attenuated VACV variants should be developed, and their properties should be investigated [8,9,10].

Investigation of the immunogenic and protective properties of candidate vaccines in comparison with those of the original VACV strain is an important stage in the development of new generations of live attenuated smallpox vaccines. Well-studied inbred BALB/c or C57BL/6 mouse strains are the most frequently used animal models for these purposes [11,12,13,14,15,16,17]. This is because a mouse model is able to mimic the immune response to VACV infection seen with human smallpox vaccination. Western blot analysis has shown that total antibodies synthesized by mice in response to VACV infection recognize a spectrum of virion proteins similar to that recognized by serum antibodies of VACV-vaccinated patients [18].

Mice can be infected with VACV in many ways, including intranasal (i.n.), intradermal (i.d.), intravenous, intraperitoneal, subcutaneous, intracranial, and intracerebral inoculation [19]. In this case, i.n. inoculation mimics a natural orthopoxvirus infection, and i.d. inoculation mimics classical smallpox vaccination. The development and level of immune response are known to depend on the used VACV strain and route of its administration. In most studies, the development of an adaptive immune response has been studied in a model of mice infected with the neurovirulent Western Reserve (WR) laboratory strain of VACV that has never been used for smallpox vaccination [11,12,13,14,15,16,17]. Therefore, the development of humoral and T-cell immunity in response to infection/vaccination with a virus should be studied for each VACV vaccine strain to be used for generating attenuated variants and for the test mouse strain.

The purpose of this study was to investigate the development of humoral and cellular immune responses in BALB/c mice i.n. or i.d. infected with the VACV LIVP strain used in Russia as the first-generation smallpox vaccine.

## 2. Materials and Methods

### 2.1. Viruses and Cells

We used clone No. 14 of the Lister-Institute for Viral Preparations (LIVP) VACV strain produced earlier by serial plaque-purifications using agarose overlay [20] and cowpox virus (CPXV) strain GRI-90 [21] adapted to mice by intranasal passages. Viruses were grown and titrated in a culture of CV-1 African green monkey kidney cells as described in [22].

### 2.2. Animals

In the study, we used inbred BALB/c strain mice received from the Animal Farm of SRC VB VECTOR. Experimental animals were fed with a standard diet and water ad libitum in compliance with the veterinary regulations and the requirements for humane care and use of animals in experimental studies. Animal studies and manipulations were approved by the Bioethics Committee of SRC VB VECTOR (Protocol No. 01-04.2021).

### 2.3. Immunization of Mice with the LIVP Virus and Sample Collection

Female BALB/c mice aged 6–7 weeks (16–19 g) were i.n. or i.d. immunized with the VACV LIVP strain at a dose of 1.6 × 10^5^ plaque forming units (PFU). Administration of the virus (i.n.; 24 mice) or 30 μL saline (6 mice) was performed following inhalation anesthesia of mice with diethyl ether. Immunization (i.d.) was performed by injecting 30 μL of infectious material (24 mice) or saline (6 mice) into the pre-shaved lower back (pelvic) region using a 30 G × 0.5 inch needle (the needle was inserted, bevel up and at a shallow angle, just under the superficial layer of epidermis; a hard bleb was seen upon successful i.d. injection). Additionally, two groups (6 mice each) of untreated animals were used as controls in an experiment assessing the protective potency of immunized mice against lethal orthopoxvirus reinfection.

The humoral and cellular immune responses of mice from i.n. or i.d. immunized groups were analyzed on days 7, 14, and 29 after immunization. Six mice inoculated with saline were used for control analysis on day 0 (1 h after saline administration by one of the above methods). On days 7, 14, and 29, six mice from each group immunized with VACV LIVP were taken for analysis. Blood was sampled antemortem from the retroorbital venous sinus with a 23 G × 1.25 inch needle. Serum was produced from individual blood samples by sedimentation of formed elements using centrifugation at 1000 g for 10 min. Produced sera were thermally inactivated at 56 °C for 30 min and stored at −20 °C.

At each time point after blood sampling, mice were sacrificed by cervical dislocation, and the spleen was sterilely removed from each of six mice from both experimental groups.

### 2.4. Splenocyte Isolation 

Spleens collected from immunized mice were mashed onto 70 μm and 40 μm cell strainers (BD Falcon™, Tewksbury, MA, USA). Splenocytes were treated with red blood cell lysis buffer (ACK Lysis Buffer, Sigma, St. Louis, MO, USA); then, cells were washed with completed RPMI 1640 medium and suspended in completed RPMI 1640 medium with 10% fetal bovine serum, 2 mM L-Gln, and 50 mkg/mL gentamycin. Cells counted with TC20™ Automated Cell Counter (Bio-Rad, Hercules, CA, USA).

### 2.5. IFN-γ ELISpot Assay

The assays were performed using the mouse IFN-γ ELISpot kit (R&D Systems, Inc., Minneapolis, MN, USA) according to the manufacturer’s instructions. Splenocytes were plated (100 µL/well) in duplicate 5 × 10^6^ cells/mL and stimulated by a mixture of peptides (corresponding to VACV-specific BALB/c mice H2-d restricted epitopes): SPYAAGYDL, SPGAAGYDL, VGPSNSPTF, KYGRLFNEI, GFIRSLQTI, and KYMWCYSQV [16,23]. Pooled peptides (100 µL/well) were added at a concentration of 20 µg/mL for each peptide. Non-stimulated and concanavalin A (Con A, 5 μg/mL) stimulated splenocytes were used as negative and non-specific positive controls, respectively. After an 18-h stimulation period at 37 °C in 5% CO_2_, cells were discarded, and plates were incubated for 2 h at 37 °C with anti-IFN-γ detection antibodies.

Plates were washed and the spots were revealed by adding the streptavidin-conjugated alkaline phosphatase and BCIP/NBT (5-bromo-4-chloro-3′-indolylphosphate/nitro-blue tetrazolium) substrate. The reaction was stopped by washing plates with distilled water. The number of IFN-γ-producing cells was counted using an ELISpot reader (Carl Zeiss, Jena, Germany).

### 2.6. Intracellular Cytokine Staining (ICS) Assays

ICS was performed according to a standard protocol as previously described [24]. Briefly, splenocytes isolated from mice 2 × 10^6^ cells/well were plated in 24-well plates and stimulated with mix of VACV-specific peptides (SPYAAGYDL, SPGAAGYDL, VGPSNSPTF, KYGRLFNEI, GFIRSLQTI, and KYMWCYSQV) or with PMA (phorbol myristate acetate, 30 ng/mL) and ionomycin (1 µg/mL). Each peptide was added at the concentration of 20 µg/mL per well, and cells were incubated for 4 h at 37 °C in 5% CO_2_ and for an additional 16 h with Brefeldin A (5 μg/mL, GolgiPlug BD Biosciences). On the next day, cells were stained with pre-titrated anti-CD3 MCA500SBB700 (BIO-RAD) anti-CD8 FITC (BD Pharmingen, San Diego, CA, USA) and anti-CD4 PerCP (BD Pharmingen, San Diego, CA, USA), fixed, and permeabilized using Cytofix/Cytoperm solution (BD Biosciences, Franklin Lakes, NJ, USA), according to the manufacturer’s instructions. Cells were then stained for intracellular cytokine detection with anti-IFN-γ APC and PE Rat Anti-Mouse IL-2 (BD Pharmingen, San Diego, CA, USA). Samples were analyzed on a ZE5 flow cytometer (Bio-Rad). Data were presented as the median and range of variation. Statistical analysis was performed using GraphPad Prism 8.0.1 software. The confidence level was calculated using the nonparametric Mann–Whitney U-test and one-way Kruskal–Wallis analysis of variance.

### 2.7. Assessment of Lethality of Cowpox Virus

BALB/c mice aged 10–11 weeks and weighing 20–23 g (6 animals per group) were i.n. infected with 10-fold dilutions of a CPXV GRI-90 preparation; survival of mice in groups was followed for 14 days, and the 50% lethal dose (LD_50_) was calculated using the Spearman–Karber method [25]. The number of lethal CPXV GRI-90 virus doses in the experiment on evaluation of the protective potency of studied VACV strain LIVP was additionally determined by i.n. inoculation (30 μL/mouse) of non-immunized mice (6 animals per dose) weighing 20–23 g with 10-fold dilutions (1/10, 1/100, 1/1000) of the CPXV GRI-90 dose (2 × 10^6^ PFU/mouse) administered to mice immunized with LIVP. The administered dose calculated by the Spearman–Karber formula was 46 LD_50_.

### 2.8. Assessment of the Protective Potency in Immunized Mice

On day 30 of the experiment, the groups of virus-immunized and control animals were i.n. infected with CPXV GRI-90 at a dose of 46 LD_50_. The animals were followed for clinical signs of infection and mortality for 14 days. Disease symptoms were assessed using the following scoring system: 0, no symptoms; 1, slightly rough hair coat; 2, rough hair coat; 3, rough hair coat and hunched posture or conjunctivitis; 4, dyspnea or lack of movement; 5, death.

Mice were individually weighed every two days. The arithmetic mean body weight of mice in each group at every time point was calculated and expressed as a percentage of the initial weight. Data scattering around the mean is presented as the standard deviation and is also expressed as a percentage.

Data were obtained for groups of six animals i.n. or i.d. immunized with VACV LIVP as well as non-immunized and not infected (negative control) or infected with CPXV GRI-90 (positive control) groups of mice.

### 2.9. Enzyme-Linked Immunosorbent Assay of Mouse Serum

An enzyme-linked immunosorbent assay (ELISA) of individual mouse sera was performed as described in [26]. A preparation of VACV LIVP virions purified by centrifugation through a sucrose pad was used as an antigen. All test mouse serum samples were titrated with two-fold serial dilutions, from a 1:100 dilution to a 1:12,800 dilution. The titration was repeated during ELISA the next day. IgM and IgG titers were determined using solutions with anti-mouse IgM and anti-mouse IgG peroxidase conjugates (Sigma, St. Louis, MO, USA), respectively. IgM and IgG titers for each test serum sample were calculated for each repeat separately and then averaged. The logarithm of the geometric mean reciprocal titer of VACV-specific IgG or IgM was calculated for experimental groups, and the confidence intervals were calculated for a 95% matching between each sample and the total population.

### 2.10. Statistics

Statistical processing and comparison of data were performed by standard methods using the Statistica 10.0 software package (StatSoft Inc., Tulsa, OK, USA 1984–2001) and GraphPad Prism 8.0.1 software package (GraphPad Software, San Diego, CA, USA). Given the binomial distribution of data from the experiment assessing the protective potency of preparations and due to the impossibility of confirming the normal distribution of experimental data, statistical analysis of the differences between the experimental and control groups, taking into account the small sample size (*n* = 6 for each group), was performed using the nonparametric Fisher’s exact test [25].

## 3. Results

### 3.1. Intranasal Infection of Mice with VACV LIVP Induces a Higher Level of Antibody Synthesis Compared to Intradermal Administration of the Virus

**Figure 1:**
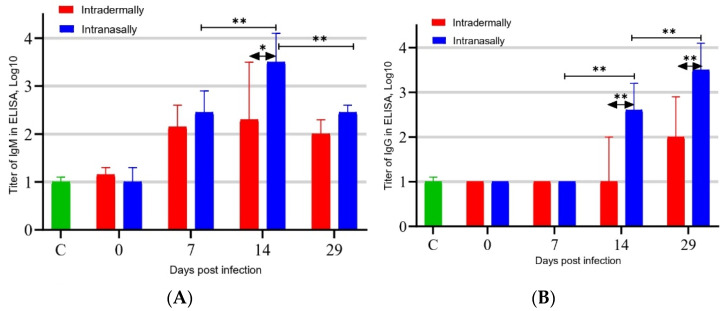
ELISA-determined concentration of VACV-specific IgM (**A**) and IgG (**B**) in blood sera of mice immunized with VACV LIVP at a dose of 10^5^ PFU. C—Control (blood sera of mice injected with saline). * *p* < 0.05; ** *p* < 0.01 calculated with the nonparametric Mann–Whitney U-test.

To assess the development of the humoral immune response in BALB/c mice to infection with the VACV LIVP virus, an infection dose of 1.6 × 10^5^ PFU/30 μL/mouse was used. The virus was inoculated intranasally (i.n.) or intradermally (i.d.) into the pelvic region (see Section 2.3) of the mouse. On days 7, 14, and 29 after infection, blood was sampled from six mice in each group immunized with VACV LIVP, and individual sera were produced. The levels of VACV-specific IgM and IgG in each individual serum were determined by ELISA, and the logarithm of the geometric mean reciprocal titer was calculated for each group at each time point. The results of these measurements, presented in Figure 1A, demonstrate that active synthesis of VACV-specific IgM in mice was observed on day 7 after infection, reached a maximum on day 14, and decreased by day 29. In this case, i.n. administration of the virus stimulated significantly higher synthesis of IgM by day 14 in comparison with i.d. inoculation of mice with VACV at the same dose (*p* < 0.05).

Synthesis of virus-specific IgG was detected only on day 14 and significantly increased by day 29 after infection of mice (Figure 1B). In this case, i.n. administration resulted in significantly higher production of VACV-specific IgG compared to i.d. administration of the virus on both day 14 and day 29 of the experiment (*p* < 0.01).

### 3.2. Infection of Mice with VACV LIVP Induces Significant Production of T-Lymphocytes

T cell responses in IFN-γ ELISpot assays were measured using splenocytes obtained on days 7, 14, and 29 after immunization of BALB/c mice with VACV LIVP. Splenocytes were stimulated by a mixture of peptides that corresponding to VACV-specific H-2d-restricted T cell epitopes: A52R_75-83_, F2L_26-34_, E3L_140-148_, C6L_74-82_, and B2R_49-57_ [16].

**Figure 2:**
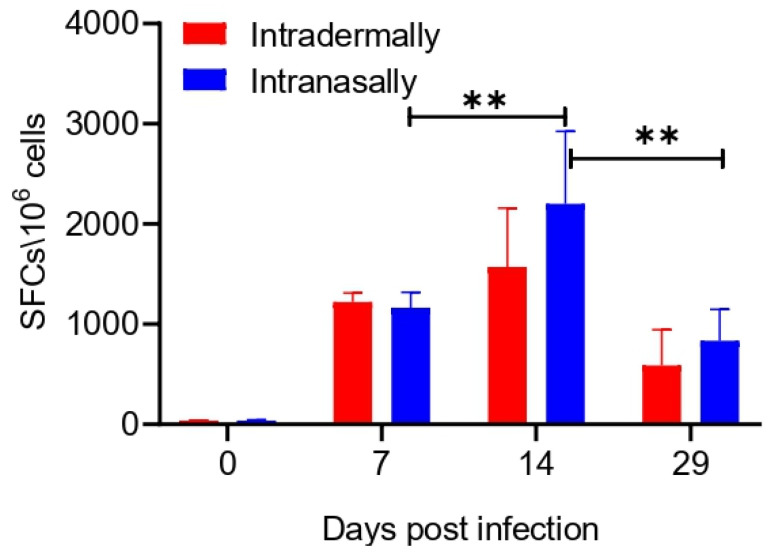
Evaluation of the T cell response in VACV LIVP intradermally or intranasally immunized BALB/c mice (n = 6 per group) using the IFN-γ ELISpot assay. Spleens were collected on days 0, 7, 14, and 29. Splenocytes were stimulated with a pool of virus-specific peptides for 24 h. Each bar represents mean of IFN-γ spot forming cells (SFCs) per million stimulated splenocytes. Data are presented as the median and range of variation. Statistical analysis was performed using GraphPad Prism 8.0.1 software. ** *p* < 0.01 calculated with the nonparametric Mann–Whitney U-test.

The assay results, presented in Figure 2, show that upon stimulation by a set of the VACV-specific peptides immunodominant for BALB/c mice, splenocytes of animals immunized by both methods secreted IFN-γ on days 7, 14, and 29 of the experiment, while splenocytes from control animals on day 0 (1 h after saline administration to mice) did not produce IFN-γ. These data indicate a significant T cell specific response in immunized mice. The response was higher upon i.n. administration than upon i.d. inoculation. The maximum release of IFN-γ by the cells was observed on day 14 in groups upon both i.n. and i.d. administration. By day 29 of the experiment, the level of VACV-specific T lymphocytes in the spleen of mice significantly decreased upon both methods of immunization (Figure 2). There were no significant differences in the levels of the T cell response in mice after i.n. or i.d. VACV administration at any time point.

### 3.3. Administration of VACV LIVP Induces Higher CD8+ T Lymphocyte Production in Comparison with CD4+ T Cell Production

**Figure 3:**
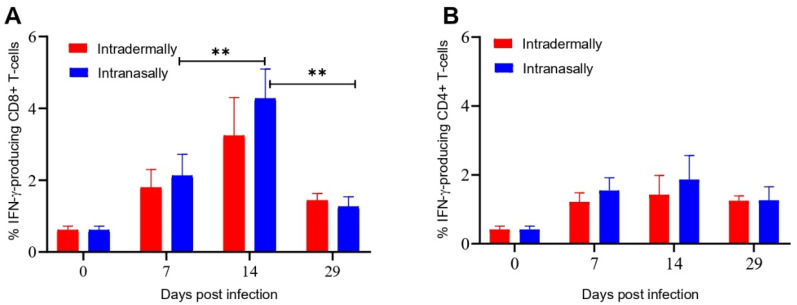
Evaluation of the level of IFN-γ producing CD8+ and CD4+ T cells from the spleen of BALB/c mice immunized with VACV LIVP using intracellular cytokine staining and flow cytometry. Spleens were collected from animals on days 0, 7, 14, and 29 after immunization. Splenocytes were stimulated with a pool of the VACV-specific peptides. Graphic representation of data in (**A**), indicating the frequency of IFN-γ producing CD8+ T cells, (**B**)The frequency of IFN-γ producing CD4+ T cells, in the spleens of mice. Data are presented as the median and range of variation. Statistical analysis was performed using GraphPad Prism 8.0.1 software. **—*p* < 0.01 calculated with the nonparametric Mann–Whitney U-test.

Intracellular cytokine staining (ICS) and flow cytometry were used to evaluate the amount of IFN-γ producing CD8+ and CD4+ cells in response to VACV-specific stimulation in populations of splenocytes isolated from the spleens of mice on different days after i.n. or i.d. infection/immunization with VACV LIVP. The CD8+ T cell response to i.n. and i.d. administration of LIVP was effectively detected on days 7 and 14 after infection and significantly decreased by day 29 (Figure 3A). In the case of CD4+ T cell response, there was no significant difference between days 7, 14, and 29 of the experiment (Figure 3B). Significant differences in the levels of CD8+ and CD4+ T cell responses to i.n. or i.d. VACV administration were not observed at any time point.

**Figure 4:**
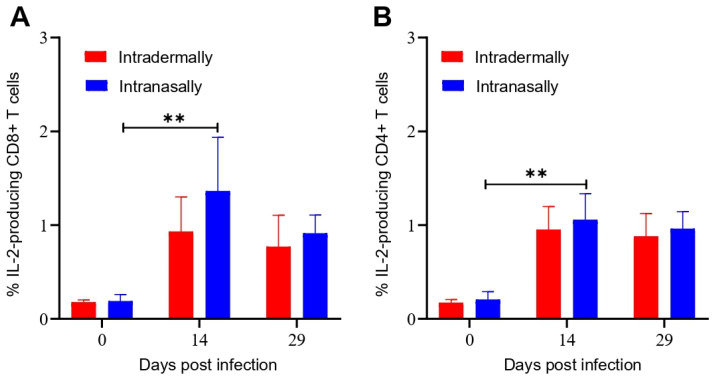
Evaluation of the level of IL-2 producing CD8+ and CD4+ T cells from the spleen of BALB/c mice immunized with VACV LIVP using intracellular cytokine staining and flow cytometry. Spleens were collected from animals on days 0, 7, 14, and 29 after immunization. Splenocytes were stimulated with a pool of the VACV-specific peptides. Graphic representation of data in (**A**), indicating the frequency of IL-2 producing CD8+ T cells, (**B**)—The frequency of IL-2 producing CD4+ T cells in the spleens of mice. Data are presented as the median and range of variation. Statistical analysis was performed using GraphPad Prism 8.0.1 software. ** *p* < 0.01 calculated with the nonparametric Mann–Whitney U-test.

The amounts of CD8+ and CD4+ T cells producing IL-2 in the populations of mice splenocytes were additionally analyzed by ICS on days 14 and 29 after VACV LIVP infection. Levels of CD8+ and CD4+ T cells producing IL-2 for both immunization procedures were comparable (Figure 4).

### 3.4. Intranasal Inoculation with VACV LIVP Provides Greater Protective Potency Than Intradermal Infection with This Virus

To elucidate how the level of humoral and cellular immune responses to vaccination of mice with VACV LIVP affects the protection level of mice from lethal orthopoxvirus reinfection, 30 days after i.n. or i.d. inoculation with the LIVP virus, mice were i.n. infected with CPXV GRI-90 at a dose of 46 LD_50_. The animals were followed for clinical signs of infection and mortality for 14 days. The mice were weighed every two days to determine changes in body weight.

**Figure 5:**
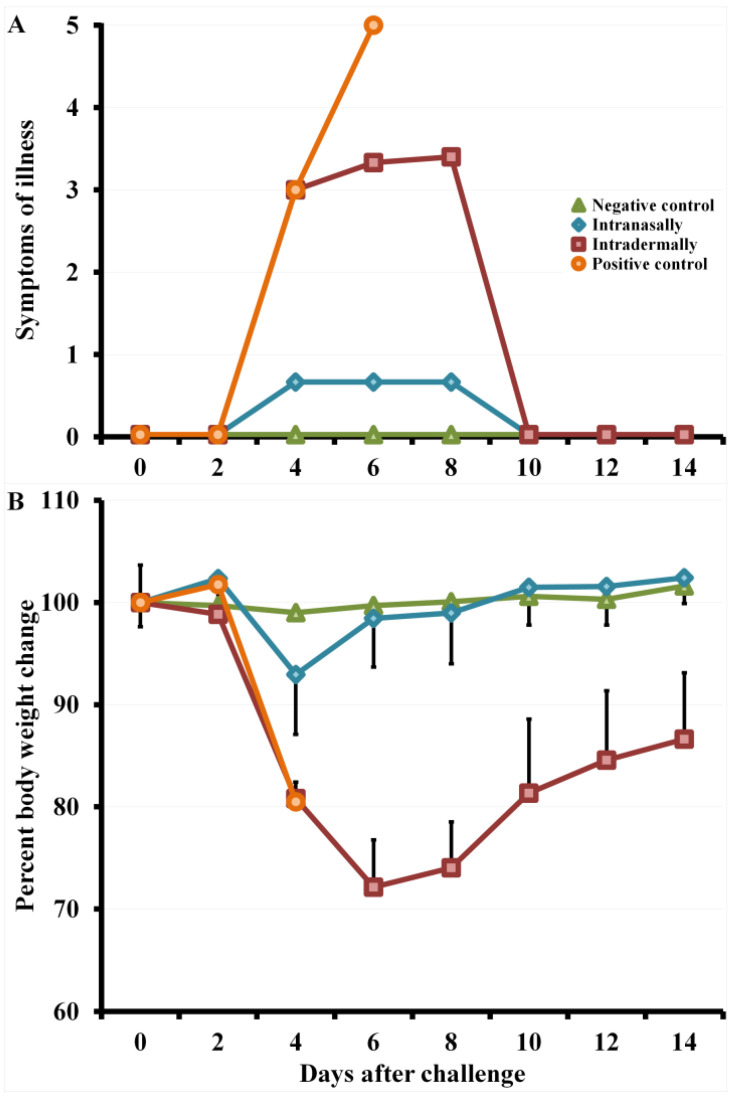
Changes in clinical signs of infection (**A**) and body weight (**B**) in mice immunized with the LIVP virus at a dose of 10^5^ PFU after intranasal reinfection with CPXV GRI-90 at a dose of 46 LD_50_ on day 30. Data are given for groups of six animals immunized with appropriate viruses as well as non-immunized and not infected (negative control) or infected with CPXV GRI-90 (positive control) groups.

**Figure 6:**
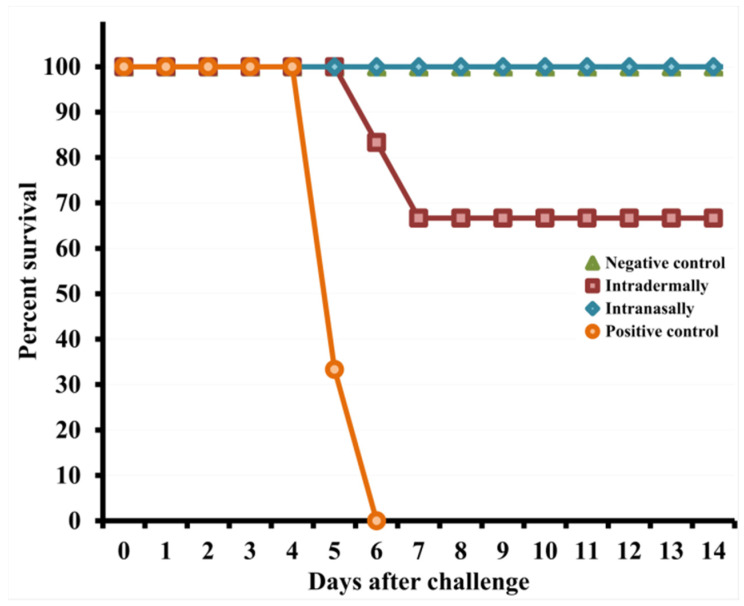
Changes in the mortality of mice immunized with the LIVP virus at a dose of 10^5^ PFU after their intranasal reinfection with CPXV GRI-90 at a dose of 46 LD_50_ on day 30 of the experiment.

On days 4–8 after infection with CPXV, mice i.d. immunized with LIVP developed severe clinical signs of the disease (Figure 5A), which were accompanied by a significant decrease in body weight (Figure 5B) and death of 33% of the animals on days 6–7 (Figure 6).

Data are given for groups of six animals immunized with appropriate viruses as well as non-immunized and not infected (negative control) or infected with CPXV GRI-90 (positive control) groups.

Some mice i.n. vaccinated with LIVP, 4–6 days after challenge with the CPXV virus, had weak signs of the disease (Figure 5A) and only a minimal short-term decrease in body weight (Figure 5B). In this case, all animals were protected from CPXV infection at a dose of 46 LD_50_ (Figure 6).

## 4. Discussion

Mass smallpox vaccination has played an important role in the eradication of smallpox. Various vaccinia virus (VACV) strains have been used as a live vaccine in different countries. The origin of these strains in most cases was not known. These VACV strains differed in pathogenicity towards different laboratory animals and in reactogenicity exhibited upon vaccination of humans [2,3,9,27].

Different VACV strains constitute the *Vaccinia virus* species and are part of the *Orthopoxvirus* genus of the *Poxviridae* family comprising the largest DNA-containing mammalian viruses. Depending on the species, the viral genome of orthopoxviruses comprises 190–220 thousand base pairs and encodes about 200 proteins. More than 90 different viral proteins are involved in structurally complex orthopoxvirus virions [28]. Most viral proteins and the complex network of their interactions with each other and proteins of the cell/organism underlie insufficient understanding of how genetic differences in VACV strains affect the dynamics and level of humoral and cellular immune responses during infection of laboratory animals [28,29].

The pathogenicity and immunogenicity of VACV depend on the used virus strain as well as the method and dose of its introduction into the animal’s body [26,30]. In most studies, the immunogenic properties of VACV variants have been studied in mice at doses ranging from 10^6^ to 10^8^ PFU [13,22,26,31]. A decrease in the immunizing dose of VACV not only reduces the reactogenicity of the virus but also decreases the level of synthesized VACV-specific antibodies [26,30].

The T cell immune response is mainly related to early proteins synthesized during VACV infection, while antibodies are largely synthesized in response to late viral proteins, both virion and some non-virion ones. It is important to note that the T cell immune response, on the one hand, and the antibody response, on the other hand, respond to different VACV antigens and cover a wide range of viral proteins [28]. In this case, the animal’s genotype determines, through the major histocompatibility complex (MHC) class I and II genes, the spectrum of numerous VACV antigens that induce an adaptive immune response after infection with this complex virus [16,32,33].

In most studies, the development of an adaptive immune response has been explored using a model of well-studied BALB/c or C57BL/6 strain mice infected with laboratory neurovirulent VACV strain WR [11,12,13,14,15,16,17]. It should be noted that this VACV strain has not been used as a smallpox vaccine and, unlike most other VACV strains, when administered to mice at high doses, leads to a lethal outcome of the infection.

The VACV LIVP strain was used in Russia as the first generation smallpox vaccine and as the basis for development of a candidate vaccine of the fourth generation [20,34]. The LIVP strain was produced by adapting the Lister strain to propagation on calf skin [2]. We have previously shown that VACV LIVP is weakly virulent for BALB/c mice, and its administration by different methods at a dose of 10^8^ PFU/mouse does not lead to a lethal outcome [26]. In this study, we investigated the development of humoral and cellular immune responses in BALB/c mice i.n. or i.d. infected with the LIVP strain at a dose of 10^5^ PFU/mouse.

On day 7 after infection, mice had active synthesis of VACV-specific IgM, which reached a maximum on day 14 and decreased by day 29. In this case, i.n. administration of the virus stimulated significantly greater synthesis of IgM by day 14 compared to i.d. infection of mice with VACV at the same dose (Figure 1A).

Synthesis of virus-specific IgG was detected only since day 14, and its level increased significantly by day 29 after infection of mice (Figure 1B). In this case, i.n. infection resulted in significantly higher production of VACV-specific IgG compared to i.d. administration of the virus.

Assessing the development of cellular immune response by the IFN-γ ELISpot assay on days 7, 14, and 29 of the experiment revealed a pronounced specific T cell response in immunized mice (Figure 2). The response was slightly higher upon i.n. administration than upon i.d. administration, and the maximum level of VACV-specific T cells occurred on day 14 in groups upon both i.n. and i.d. administration, but no significant differences in the levels of T cell response in mice after i.n. or i.d. VACV administration at any time point were revealed. By day 29 of the experiment, the level of VACV-specific T lymphocytes in the spleen of mice significantly decreased for both methods of immunization (Figure 2).

The ELISpot results correlate with the ICS data (Figure 2 and Figure 3). The number of VACV-specific IFN-γ producing CD8+ T cells increased on day 7, and the maximum was detected on day 14. By day 29, the number of CD8+ T cells decreased for both vaccination options (Figure 3A). In the case of the CD4+ T cell response, there was no significant difference between days 7, 14, and 29 of the experiment (Figure 3B). Differences in the levels of the CD8+ and CD4+ T cell responses to i.n. or i.d. VACV administration were not significant at any time point.

It should be noted that the maximal T-cell immune response after infection of BALB/c mice with the VACV LIVP virus occurred on day 14 (Figure 2 and Figure 3). Unlike the LIVP strain, the peak of the T-cell response in mice inoculated with the WR strain was observed on day 7 after infection [11]. Apparently, this delayed development of the adaptive immune response, characteristic of the LIVP strain, is due to its lower virulence for BALB/c mice compared to that of the WR strain. The fact that the level of IFN-γ production is higher than IL-2 (Figure 3 and Figure 4) indicates the formation of the virus-specific CD8+ T cells with the cytotoxic phenotype. The level of CD4+ T cells producing IFN-γ and IL-2 indicates the formation of a T-h response. At the same time, the CD8+ response was more expressed than the CD4+ response.

To elucidate how the level of humoral and cellular immune responses to vaccination of mice with VACV LIVP affects the level of protection of mice against lethal orthopoxvirus reinfection, the mice were infected with lethal CPXV GRI-90 at a dose of 46 LD_50_ 30 days after immunization with the LIVP virus.

On days 4–8 after CPXV infection, mice i.d. immunized with LIVP developed severe clinical signs of the disease (Figure 5A), which were accompanied by a significant decrease in body weight (Figure 5B) and death of 33% of the animals on days 6–7 (Figure 6).

Some mice i.n. vaccinated with LIVP displayed only mild signs of the disease (Figure 5A) and minimal weight loss (Figure 5B) on days 4–6 after challenge with the CPXV virus. In this case, all animals were resistant to lethal CPXV infection (Figure 6).

Therefore, i.n. administration of VACV LIVP to mice induced a significantly higher level of IgG production compared to i.d. injection (Figure 1B) and, thereby, provided complete protection of the mice from this infection (Figure 6). It should be noted that the level of the VACV-specific T-cell immune response was comparable for i.n. and i.d. immunization with the LIVP virus (Figure 3 and Figure 4) under the conditions of our experiments. These data allow us to draw a conclusion that coincides with the previous opinion of different authors that the level of the humoral immune response to vaccination plays a decisive role in protecting animals from orthopoxvirus reinfection [13,30,35]. The cellular immune response undoubtedly also plays an important role in protecting against orthopoxvirus reinfection [35].

## Data Availability

All raw data are available and provided upon request.
